# Cancer diagnosis in the post-coronavirus disease era: the promising role of telepathology and artificial intelligence

**DOI:** 10.1590/1806-9282.2024S127

**Published:** 2024-06-07

**Authors:** Clóvis Klock, Fernando Augusto Soares

**Affiliations:** 1 Brazilian Society of Pathology (President 2023-2024), Department of Diagnostic Medicine – São Paulo (SP), Brazil.; 2 Medicina Diagnóstica Ltda – Erechim (RS), Brazil.; 3 D'Or IDOR Network Research Institute, Department of Pathology – São Paulo (SP), Brazil.; 4 Universidade de São Paulo, Faculty of Dentistry, Department of Stomatology – São Paulo (SP), Brazil.

## INTRODUCTION

Cancer is one of the main public health challenges worldwide, being one of the leading causes of death and representing a significant barrier to increasing life expectancy. In many countries, cancer is the first or second leading cause of premature death before the age of 70. Cancer incidence and mortality are on the rise worldwide^1^. This increase is a result of demographic and epidemiological transitions taking place globally. From a demographic perspective, there is a reduction in the fertility rate and infant mortality, resulting in an increase in the proportion of elderly people in the population. The epidemiological transition, on the other hand, reflects the gradual shift from mortality from infectious diseases to deaths related to chronic diseases. Population aging and changes in behavior and environment, such as structural changes affecting mobility, recreation, diet, and exposure to environmental pollutants, contribute to increased cancer incidence and mortality^2^.

In countries with a high human development index (HDI), impacts on incidence and mortality rates have been observed through effective actions for the prevention, early detection, and treatment of cancer. On the contrary, in countries in transition, these rates continue to increase or, at most, remain stable. The challenge for less developed countries is to make more effective use of available resources and efforts to control cancer.

According to estimates by the Global Cancer Observatory (Globocan), prepared by the International Agency for Research on Cancer (IARC), in 2020, there were about 19.3 million new cases of cancer worldwide (excluding cases of non-melanoma skin cancer, which totaled 18.1 million). It is estimated that one in five people will get cancer in their lifetime^1,3^. The 10 most common cancers account for more than 60% of new cases. Female breast cancer is the most common cancer globally, with 2.3 million (11.7%) new cases, followed by lung cancer, with 2.2 million (11.4%); colon and rectum, with 1.9 million (10.0%); prostate, with 1.4 million (7.3%); and non-melanoma skin, with 1.2 million (6.2%) new cases.

For Brazil, the estimate for the three-year period from 2023 to 2025 indicates that there will be approximately 704,000 new cases of cancer, 483,000 of which are cases of non-melanoma skin cancer when cases of non-melanoma skin cancer are excluded. Non-melanoma skin cancer is estimated to be the most prevalent, accounting for about 220,000 cases (31.3%). Next is breast cancer, with 74,000 cases (10.5%); prostate, with 72,000 cases (10.2%); colon and rectum, with 46,000 cases (6.5%); lung, with 32,000 cases (4.6%); and stomach, with 21,000 new cases (3.1%)^4^.

When analyzing the most frequent types of cancer in men, there is a predominance of non-melanoma skin cancer, with 102,000 cases (29.9%); followed by prostate cancer, with 72,000 cases (21.0%); colon and rectum, with 22,000 cases (6.4%); lung, with 18,000 cases (5.3%); stomach, with 13,000 cases (3.9%); and oral cavity, with 11,000 cases (3.2%).

In women, the most common cancers are non-melanoma skin cancers, with 118,000 cases (32.7%); breast, with 74,000 cases (20.3%); colon and rectum, with 24,000 cases (6.5%); cervix, with 17,000 cases (4.7%); lung, with 15,000 cases (4.0%); and thyroid, with 14,000 cases (3.9%)^4^.

The coronavirus disease 2019 (COVID-19) pandemic has had a profound impact on health and the global economy. As of October 2023, there were a total of 771,191,203 confirmed cases of COVID-19, with 6,961,014 deaths^5^. In the field of health, the impact was significant. The health system in several countries has been overwhelmed, with an urgent need for hospital beds, personal protective equipment, and health workers. Many hospitals and health facilities have worked beyond their maximum capacity, struggling to care for all patients affected by the disease. COVID-19 has proven to be a serious health threat, especially for vulnerable groups such as the elderly and people with pre-existing conditions. In addition to health, the pandemic has also had a devastating impact on the global economy. Business closures, travel restrictions, and lockdown measures have resulted in a collapse in tourism, retail, entertainment, and many other sectors. Millions of people lost their jobs and faced financial hardship. Governments around the world have had to take urgent action to contain the impact on the economy by implementing financial stimulus packages, aid programs, and support for businesses. Despite these efforts, economic recovery has been an ongoing challenge, with long-lasting consequences for many industries and individuals. Vaccination has been a key tool in the fight against the disease. As of October 5, 2023, a total of 13,516,185,809 vaccine doses had been administered. Mass vaccination was a hope to control the spread of the virus, lessen the severity of the disease, and reduce the number of deaths^5^.

In May 2020, the American Society of Clinical Oncology (ASCO) published a special report recommending the postponement of any clinic visits and any cancer screening, diagnosis, or staging-related procedures if this postponement does not pose a risk of disease progression or worsening prognosis^6^. Some international studies show that the decrease in cancer diagnoses in the first months of the pandemic was 65.2% of new cancer cases^6^. Screening for some cancers has been hampered, with data showing that breast, colon, and rectal cancers were the most affected, with 89.2 and 84.5%, respectively. In a study carried out in the United Kingdom, the lockdown caused the suspension of cancer screenings, compromising the early diagnosis of numerous patients. Only in this case were patients with critical and symptomatic clinical conditions directed to diagnostic intervention. Cancer records from the National Health Service (NHS) were used through hospital databases with patients aged 15–84 years diagnosed with breast cancer (35583), colorectal cancer (24,975), and esophageal cancer (6,744) in 2010 with follow-up until 2014. In patients with primary lung cancer (29,305), 2012 was used as the year of diagnosis and 2015 as the final follow-up date. Using a flowchart to define the pathways of cancer patients within the NHS, an estimate was made to assess the consequences of delayed diagnosis in this group of patients over a period of 12 months, starting in March 2020 (lockdown date), contextualizing its impact 1, 3, and 5 years after the initial diagnosis. In this methodology, three pathways or flows of these patients were considered, corresponding to the best to the worst scenario. Based on this, the actual impact of survival at 1, 3, and 5 years after diagnosis was estimated, thus calculating the total number of deaths attributed to cancer and the total number of years of life lost compared with pre-pandemic data^7^.

In Brazil, there are several articles reporting the impact of the pandemic on anatomical and pathological diagnoses of cancer, especially in the public health system. The Brazilian Society of Pathology (SBP) was one of the first societies to warn about the problem of cancer diagnosis in the midst of the pandemic. In an article published in Folha de São Paulo on April 17, journalist Claudia Colucci interviewed several representatives of medical societies, among which Dr. Clóvis Klock, at the time President of the Advisory Board of SBP., warned that many pathology services had a 70–80% decrease in cancer diagnoses at the beginning of the pandemic^8^. Subsequently, many articles have demonstrated these aspects of the prediction and impact of the decrease in diagnoses, both in Brazil and in other countries. This impact has been greater in some countries, especially in the case of the most vulnerable people^9–12^.

In all scenarios, an increase of 7.9–9.6% in breast cancer deaths was estimated within 5 years after diagnosis, meaning 281–344 more deaths, respectively. In colorectal cancer, the increase was from 15.3% (1,445) to 16.6% (1,563), and in lung cancer, the increase was from 4.8% (1,235) to 5.3% (1,372). And finally, the increase seen in patients with esophageal cancer was 5.8% (330) to 6% (342). These data show that there has been a significant increase in preventable deaths in the United Kingdom, likely due to restrictive measures and social isolation^8^. Another study^13^ observed a 40% reduction in the weekly incidence of cancer in the Netherlands and 75% in the United Kingdom since the beginning of the COVID-19 pandemic. This study used a methodology similar to ours, evaluating the records in a database from January to April 2019 comparing them with the same period in 2020.

Delays in cancer diagnosis can occur at different levels of health care: the patient level, primary care, and secondary care. Late diagnoses of more advanced neoplastic diseases may occur when patients are slow to recognize and act on suspicious symptoms^6^. Lack of awareness about early cancer symptoms is the main reason for late presentation, especially when symptoms are atypical^6^. In addition, the high demand for specialized medical services can create an additional barrier, delaying diagnosis, especially in public health services^4^.

The COVID-19 pandemic has had significant impacts on cancer diagnosis and treatment, with delays in detection and overburdening health systems. In this context, telepathology and artificial intelligence (AI) emerge as promising tools to overcome these challenges and provide accurate and timely diagnoses^14^. Telepathology allows the remote analysis of pathological samples, especially slides, whether hematoxylin and eosin, or special techniques, such as immunohistochemistry, facilitating access to specialists and collaborative interpretation of complex cases^15^. With telepathology, it is possible to send scanned images of slides to specialists anywhere in the world, allowing for accurate and rapid assessment. This is especially relevant in resource-constrained areas or during public health crises such as the COVID-19 pandemic^14^. Telepathology can be used in several stages of cancer diagnosis, including screening, primary diagnosis, and second opinion, providing greater agility and access to specialized care. AI, through advanced algorithms, can analyze large amounts of data quickly and accurately. In cancer diagnosis, AI has shown promising results in early detection, differentiation between benign and malignant lesions, classification of cancer subtypes, and selection of personalized therapies. These capabilities can help speed up the diagnostic process and improve accuracy, allowing for more appropriate and timely treatment for patients^16–18^.

The use of telepathology and AI in cancer diagnosis can bring several benefits to overcoming the challenges posed by the COVID-19 pandemic. These technologies make it possible to carry out remote consultations, avoiding the need for patients to travel and reducing the risk of contamination^14^. In addition, AI's ability to analyze quickly and accurately contributes to decreasing diagnostic delays and providing reliable results. Implementing these technologies can improve access to healthcare services, particularly in remote or resource-limited areas^18^.

The use of telepathology and AI in cancer diagnosis raises important ethical and regulatory considerations. Resolution No. 2,264/2019^19^ regulates the use of telepathology in Brazil. It is necessary to ensure the privacy and protection of patient data, informed consent for the use of technologies, and the appropriate regulation of companies that develop and market telepathology and AI solutions, following the General Data Protection Law (Law No. 13,853) of 2019^20^. In addition, it is essential to ensure that these technologies are used as an auxiliary tool for physicians, respecting the expertise and clinical judgment of healthcare professionals.

Cancer diagnosis faces significant challenges in the context of the COVID-19 pandemic. Telepathology and AI emerge as promising solutions for early detection and accurate diagnosis, overcoming delays and reducing the need for patients to travel. The implementation of these technologies requires appropriate ethical and regulatory considerations to ensure their responsible and effective use. Going forward, telepathology and AI are expected to continue to evolve, providing significant advancements in cancer diagnosis and treatment, regardless of public health crises like COVID-19.

In addition, it is important to highlight that telepathology and AI can also be useful in the monitoring and follow-up of cancer patients, enabling the early identification of recurrences and the adjustment of treatments in a personalized way. These technologies have the potential to revolutionize the approach to cancer by offering more accurate, efficient, and accessible medicine. Therefore, investments in research, development, and implementation of telepathology and AI in the context of cancer are essential to improve treatment outcomes and quality of life for patients.

Telepathology and AI are promising tools in cancer diagnosis, especially in the post-COVID-19 pandemic context. These technologies can provide accurate and timely diagnoses, overcoming delays caused by social distancing measures and overburdening healthcare services. However, it is critical to ensure the protection of patient data, proper regulation, and responsible use of these technologies. With continued investments and advancements, telepathology and AI are expected to play a crucial role in improving access to healthcare services and optimizing cancer diagnosis and treatment, achieving better outcomes for patients worldwide.
